# Challenges of diagnosing rare cancers in primary care

**DOI:** 10.3399/bjgp24X738789

**Published:** 2024-07-26

**Authors:** Sarah F Moore, Gary A Abel, Luke TA Mounce

**Affiliations:** Department of Health and Community Sciences, University of Exeter, Exeter.; Department of Health and Community Sciences, University of Exeter, Exeter.; Department of Health and Community Sciences, University of Exeter, Exeter.

Rare cancers might only have an annual incidence of less than 6 in 100 000 but taken together they make up nearly a quarter (24%) of all cancers diagnosed each year.^1^ This includes more than 200 types of cancer and not only considers those at rare sites, for example, gallbladder, but also rare subtypes in a common cancer location, for example, inflammatory breast cancer. Indeed, the percentage of rare cancers may well be higher than 24%, as we should also consider common cancers of a common subtype in an unusual setting, such as, colorectal cancer in an otherwise healthy 29-year-old.

Diagnosing all cancers earlier is a priority for patients and the public, with the NHS Long Term Plan aiming to increase the percentage of cancers diagnosed at an early stage from current levels of around 50% up to 75% by 2028.^2^ This is even more important for rare cancers where only around 40% are diagnosed at an early stage.^3^ This contributes to the reduced 5-year survival in patients with rare (49%) compared to common (63%) cancers.^4^ However, diagnosing rare cancers is fraught with difficulties.

## What are the barriers to diagnosing rare cancers?

In medical school, doctors are taught to look for horses when we hear hoofbeats, but GPs must always be aware of the possibility that a zebra might be the culprit. This uncertainty and the potential misattribution of symptoms to common causes is one of the most difficult parts of being a GP and one of the barriers to diagnosing rare cancers. When an older person presents with backache the most likely cause is musculoskeletal, and both patient and practitioner may well collude in agreeing on this cause without considering other possibilities such as myeloma.

It is not just in primary care that these barriers occur, indeed they begin even before a patient presents to their GP, and can relate to awareness of symptoms and recognising their severity and need to seek medical input. Then, even when onward referral does take place, diagnosis can be delayed waiting for specialist input or due to the wrong type of test being performed.^5^ On a wider scale there are barriers within healthcare systems, including a tendency to underinvest in awareness campaigns or research into rare cancers.

## How can we diagnose rare cancers earlier?

The barriers we have highlighted certainly shouldn’t make us give up trying to diagnose cancers earlier, as we know that expediting diagnosis is likely to lead to benefits for patients in terms of improved survival, earlier stage diagnosis, and improved quality of life.^6^

Building an evidence base for best practice for the detection of rare conditions can be difficult. Studies focusing on rarer cancers must adopt designs that maximise the number of patients with the cancer included to have sufficient power to detect meaningful effect sizes and adequate precision on estimates. The classic cohort study, most suited to exploring the positive predictive value of cancer features, selects patients based on exposures (for example, presence of a cancer feature) and is unlikely to result in sufficient numbers with the outcome (the cancer of interest). Hence, case–control designs (where the sample is selected based on the outcome) or population-based studies are more appropriate. Even with such a design, inclusion criteria may need to be broad to attain sufficient numbers, which could introduce bias and impact the generalisability of study findings.

Small sample sizes can affect a study’s potential impact, as methods and conclusions are constrained. For example, in Rafiq *et al’s* cohort study on clinical activity and contacts in general practice prior to sarcoma diagnosis (published in this month’s *BJGP*),^7^ the authors selected a sample who all have either soft tissue or bone sarcoma and looked backwards from diagnosis to explore GPs’ clinical activity. This study maximised its sample size by selecting a cohort of cases from a whole state (Victoria) by using routinely collected health data covering a nearly 20-year period starting in 2002. A broad study window, however, could lead to the introduction of biases due to, for example, changes in data recording quality or practices, clinical policy, or demographic shifts.

**Figure 1.**
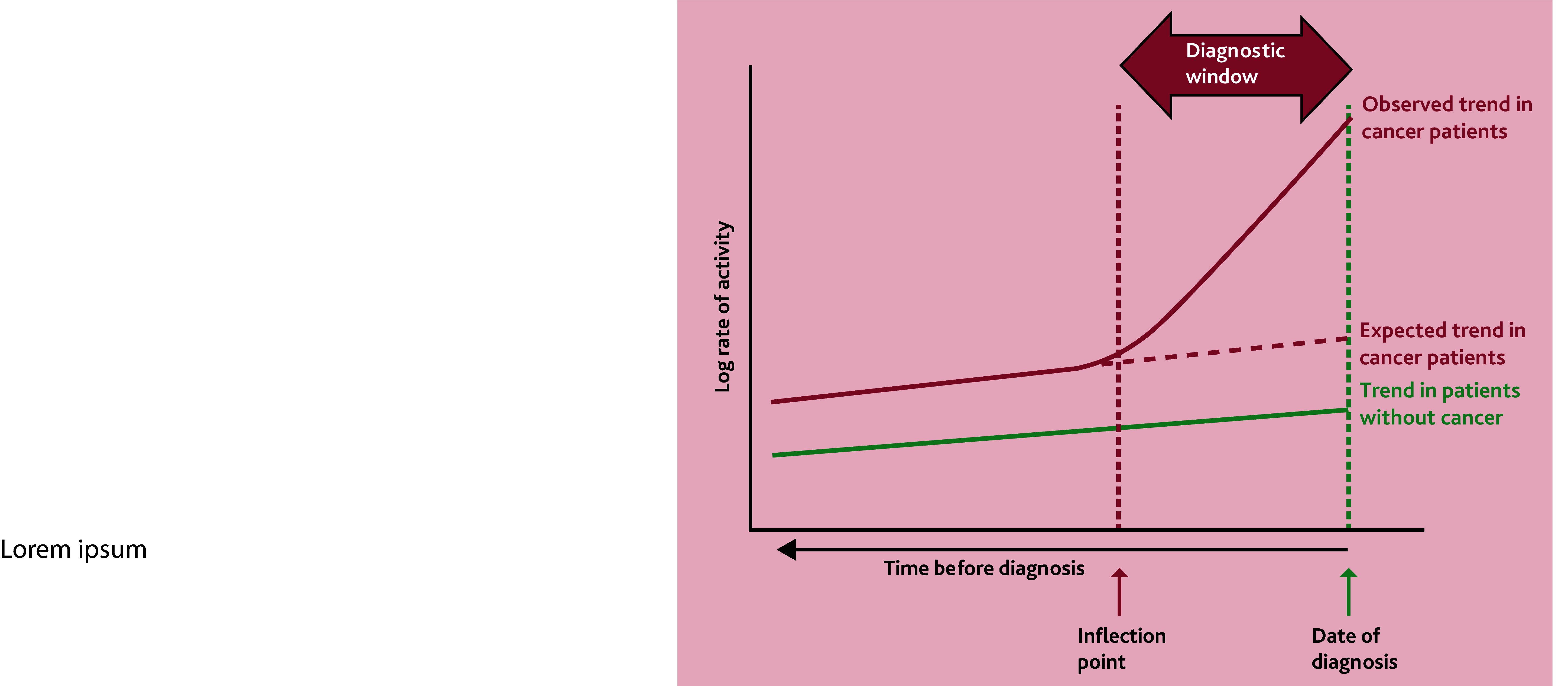
Illustration of diagnostic window studies.

The authors used recently developed methodology (see Figure 1 and Price *et al*^8^) to explore ‘diagnostic windows’ indicating where earlier cancer detection may be possible. Diagnostic window studies are used to identify changes in activity (for example, consultations, prescriptions, or imaging requests) in populations leading up to a diagnosis of a disease, in this case cancer. To estimate a diagnostic window, the data from individual patients is aligned to the date of diagnosis and the rate of the chosen activity across all patients is calculated relative to this date of diagnosis. Sometimes this is done just for patients with the disease (as in the study by Rafiq *et al* ^7^) and sometimes a matched cohort of control patients is used where the diagnosis date of the matched cancer patient is adopted by the control. Background rates of activity (in both cancer and control patients) are often seen to increase over time due to the population ageing. Background activity rates are also often higher in cancer patients than controls due to higher prevalence of risk factors that are common between cancer and other conditions (for example, cardiovascular disease). The time point at which the observed rates of activity in patients with cancer deviate from that expected based on the background trend, known as the inflection point, is then identified. The diagnostic window is defined as the time between this inflection point and the date of diagnosis. It is important to remember that diagnostic windows describe what is happening for a population, rather than for individuals, and so we only know that activity is increasing for at least some cancer patients at the inflection point, and is unlikely to be increasing for all patients. These analyses offer a robust alternative to problematic patient-level metrics of diagnostic timeliness, and can highlight subtle signals in medical records of people who are subsequently diagnosed with cancer. The principal finding in the Australian sarcoma study was that rates of imaging requests increased 6 months pre-diagnosis, before rates of GP consultations began to increase. More work is needed to understand what triggered early imaging requests and why these did not lead to prompter diagnosis.

## Future directions

There are clearly big challenges involved in diagnosing rare cancers and improving diagnosis is going to require disruption of pathways, not just improving those that already exist.

Other options for aiding doctors to think about rare cancers include the development and use of clinical prediction tools that can gather data from the GP record and display prompts to consider the diagnosis. Producing these for rare cancers will be a challenge given the numbers needed to develop and validate the tools.^9^ There is also great scope for improving education of both the public and healthcare professionals, and we should also work together to empower patients and carers to challenge medical professionals.

One example, which highlights the potential of collaboration, is the International Rare Cancers Initiative, which combines the forces of cancer research networks across multiple countries, with the aim of improving treatments for patients with rare cancers by facilitating the development of international clinical trials.^10^^,^^11^ Perhaps in future, similar consortia could begin to tackle the challenges of improving early diagnosis.
